# High-sensitivity acoustic sensors from nanofibre webs

**DOI:** 10.1038/ncomms11108

**Published:** 2016-03-23

**Authors:** Chenhong Lang, Jian Fang, Hao Shao, Xin Ding, Tong Lin

**Affiliations:** 1 College of Textiles, Donghua University, Shanghai 201620, China; 2 Institute for Frontier Materials, Deakin University, Geelong, Victoria 3216, Australia

## Abstract

Considerable interest has been devoted to converting mechanical energy into electricity using polymer nanofibres. In particular, piezoelectric nanofibres produced by electrospinning have shown remarkable mechanical energy-to-electricity conversion ability. However, there is little data for the acoustic-to-electric conversion of electrospun nanofibres. Here we show that electrospun piezoelectric nanofibre webs have a strong acoustic-to-electric conversion ability. Using poly(vinylidene fluoride) as a model polymer and a sensor device that transfers sound directly to the nanofibre layer, we show that the sensor devices can detect low-frequency sound with a sensitivity as high as 266 mV Pa^−1^. They can precisely distinguish sound waves in low to middle frequency region. These features make them especially suitable for noise detection. Our nanofibre device has more than five times higher sensitivity than a commercial piezoelectric poly(vinylidene fluoride) film device. Electrospun piezoelectric nanofibres may be useful for developing high-performance acoustic sensors.

## Abstract (web summary)

Polymer nanofibres can be used to detect mechanical motion. Here, the authors use electrospun piezoelectric nanofibre webs to detect acoustic waves at frequencies below 500 Hz with a good sensitivity at low pressure levels, and study the impact of the fibres morphology and crystalline phase.

## Graphical abstract

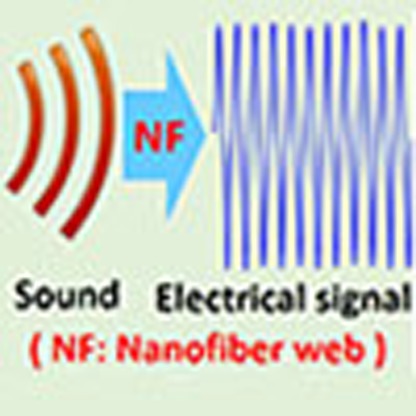

Detection of sound, an acoustic wave, has been applied in many fields including environment protection^1^, industrial manufacture^2^, healthcare^3^, medical^4^, scientific research^5^ and defence^6^. Numerous acoustic sensors have been developed based on various transducing principles such as piezo-resistance^7^, piezo-capacitance^8^, piezo-optics^9^ and piezoelectricity^10^,^11^,^12^. Piezoelectric acoustic sensors especially for those made of piezoelectric polymers are distinct from the others in that they have higher sensitivity and flexibility and can be processed into diverse geometries (for example, flat or non-flat film)^10^,^11^,^12^. However, most of the piezoelectric polymers need to be processed by a tedious multistep method involving mechanical stretching and high-voltage poling at an elevated temperature to attain the piezoelectricity before making transducers^13^, which are also of energy consumption and high cost.

Recently, the piezoelectricity of nanofibres prepared by electrospinning have been reported^14^,^15^,^16^,^17^,^18^. Our group and other researchers' studies have indicated that nanofibres electrospun from piezoelectric polymers, such as poly(vinylidene fluoride) (PVDF), poly(vinylidene fluoride-*co*-hexafluoropropene) (PVDF-HFP) and poly(vinylidene fluoride-*co*-trifluoroethylene) (P(VDF-TrFE)), have strong piezoelectricity without stretching and poling treatments^16^,^17^,^18^,^19^,^20^,^21^,^22^,^23^,^24^. These piezoelectric nanofibres have shown great potential for making mechanical sensors and energy generators^16^,^18^,^19^,^22^. The voltage outputs of randomly orientated PVDF nanofibre webs under compressing or bending are even higher than those of commercial piezoelectric PVDF films. However, little has been reported on the acoustic-to-electric energy conversion of polymer nanofibres in research literature to date.

Here we show that piezoelectric nanofibres prepared by electrospinning have an incredible acoustic-to-electric conversion ability. Using PVDF as a model polymer and a device structure that allows sound to directly transfer to the nanofibre layer, we show that the nanofibre sensor devices are able to detect low-frequency sound with a sensitivity as high as 266 mV Pa^−1^. In comparison with the sensor device made of a commercial piezoelectric PVDF dense film, our nanofibre devices have more than five times higher sensitivity. They can precisely distinguish sound waves in the low to middle frequency region. The devices show higher sensitivity to the sound of the pressure level above 100 dB, which is very suitable for detecting noise.

## Results

### Preparation of sensing material and device

**Figure 1:**
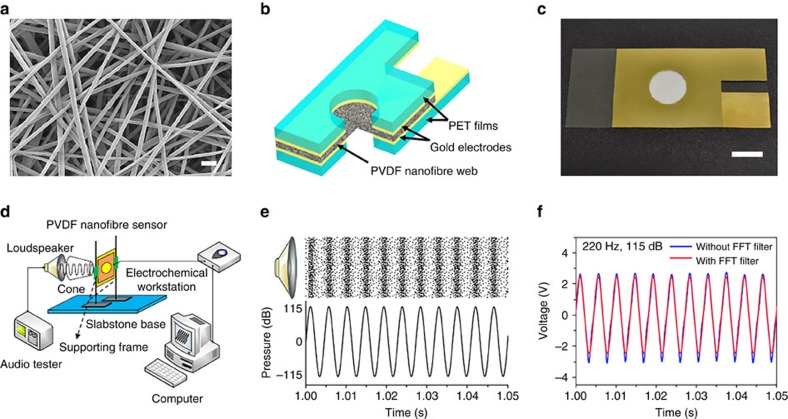
Material and characterization. (**a**) SEM image of the PVDF nanofibres (scale bar, 1 μm), (**b**) schematic illustration of sensor structure, (**c**) digital photo of the device (scale bar, 1 cm), (**d**) schematic illustration of the setup for testing the sensor device, (**e**) illustration of sound wave (the black dots illustrate the motion of air molecules associated with sound), (**f**) voltage outputs of the device under sound with and without FFT treatment (hole diameter, 12.8 mm; web thickness, 40 μm; web area, 12 cm^2^).

PVDF nanofibres were prepared by a needle electrospinning technique. Figure 1a shows the morphology of the as-electrospun fibres. All fibres looked uniform without bead. The fibres had a diameter of 310±60 nm (see the histogram of diameter distribution in Supplementary Fig. 1). The X-ray diffraction pattern and Fourier transform infrared spectrum indicated that the PVDF nanofibres mainly contained α and β crystal phases, with the β-phase content as high as 86% (see the detail calculation in Supplementary Fig. 2). These results are in good accordance with our previous findings^16^,^21^, and the high β-phase content is beneficial to mechanical-to-electronic energy conversion^25^.

The acoustic sensor was prepared by sandwiching a piece of PVDF nanofibre web with two transparent polyethylene terephthalate (PET) films (thickness 110 μm), which were gold sputter-coated on one side. The gold-coated surfaces were contacted with the nanofibre layer, and they functioned as electrodes to collect electrical signals. Figure 1b schematically illustrates the sensor structure and a photo of the actual device is shown in Fig. 1c. To allow the nanofibres to directly receive sound waves, a through hole was cut on each plastic film. Figure 1d shows the setup for measuring the sensing property. A speaker with a sound-collecting cone was used as sound source, and the narrow end of the cone faced the hole of the device. To avoid false signals generated from the vibration of the testing equipment and mechanical components other than the nanofibre web, the testing equipment and the sensor device were mounted on a heavy slabstone base. In this way, the mechanical vibrations from background, supporting base and frame had negligible contribution to the voltage output of the sensor device (see the test results in Supplementary Figs 3 and 4), even at low acoustic pressure levels (Supplementary Fig. 5).

### Acoustic sensor behaviours

When sound waves (Fig. 1e) reached the nanofibre device, electric signals generated, as shown in Fig. 1f. At the sound wave of frequency 220 Hz (intensity level 115 dB), the device generated a periodic voltage output with peak value as high as 3.10 V (also see Supplementary Fig. 6). The overall output is of a typical alternating current. To eliminate the noise signals from the background sound in the testing environment, electronic instrument and connecting cables, we used a fast Fourier transform (FFT) technique to process the output signals^26^ (see details in Supplementary Fig. 6). After FFT filtering using the sound frequency, the voltage output was very similar to the raw data (Fig. 1f). However, when the FFT filtering was conducted using the frequency beyond the sound frequencies, the output was very small (Supplementary Fig. 6). This suggests that the voltage response chiefly comes from the sound waves.

**Figure 2:**
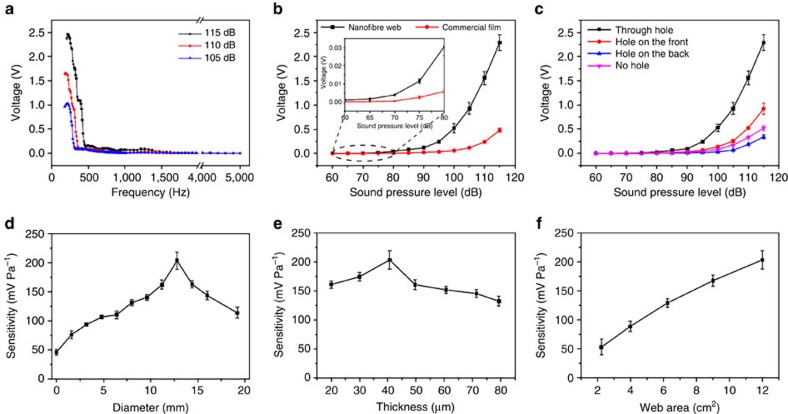
Voltage outputs and sensitivity of the sensor devices. Effect of (**a**) sound wave frequency, (**b**) SPL (sound wave frequency, 220 Hz), (**c**) hole profile on device voltage outputs. Effect of (**d**) hole diameter (web thickness, 40 μm; sound wave frequency, 220 Hz; SPL, 115 dB), (**e**) thickness of the nanofibre web (hole diameter, 12.8 mm; sound wave frequency, 220 Hz; SPL, 115 dB), (**f**) nanofibre web area (hole diameter, 12.8 mm; web thickness, 40 μm; sound wave frequency, 220 Hz; SPL, 115 dB) on device sensitivity. The error bars represent the s.d. of the test results of at least three replicates.

By maintaining the sound pressure at the same level while varying the sound frequency, the effect of sound wave frequency on voltage output was examined. As shown in Fig. 2a, the voltage output changes with sound frequency. The maximum voltage output was obtained at 220 Hz. When the frequency was above 220 Hz, a decrease in the voltage value resulted. A little fluctuation in the output was also observed in the frequency range of 400–1,500 Hz. The voltage output decreased to nearly zero when the sound frequency was over 2,000 Hz. It is known that sound is hearable typically in a frequency ranging between 20 and 20,000 Hz (refs 27, 28). The noise pollution existing widely in road traffic, airports, industrial and many public places primarily comes from the sounds of low frequency (20–200 Hz). The nanofibre device could be useful for the detection of acoustic waves in those areas.

At three acoustic pressure levels, the device showed a similar voltage output profile, except for different voltage values. This indicates that the nanofibre acoustic sensor is sensitive to low to medium frequency sound waves, and the voltage output is affected by sound pressure level (SPL). Figure 2b shows the effect of SPL on the voltage output of the device. At the same sound frequency, the output increased from 1.2 to 245 mV when the SPL changed from 60 to 95 dB. When the SPL was higher than 100 dB, the voltage output increased nearly in linear manner with increasing the SPL. When the SPL was 115 dB, the output reached 2.29 V. It is known that the sound in the SPL range of 90–110, 110–120, 120–130 and the above is ranked as ‘very noisy', ‘extremely noisy', ‘painful' and ‘intolerable' noise, respectively^29^. The large voltage response to sound pressure higher than 100 dB suggests that the nanofibre device could be useful for monitoring noise.

Sensitivity is a key parameter to sensors. The sensitivity (*S*) for acoustic sensors can be calculated by the equation (1) (refs 10, 30),


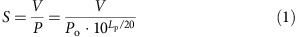


where *P* is the sound pressure, *V* is the voltage output of the device, *P*_o_ is the reference sound pressure of 0.00002 Pa and *L*_p_ is the SPL in decibel. Based on Fig. 2b, the sensitivity of the nanofibre device was estimated, being as high as 266 mV Pa^−1^. For comparison, a commercial piezoelectric PVDF film (the same dimension and thickness) was also subjected to the same test. As also shown in Fig. 2b, at the same SPL, the outputs from the dense film device are all smaller than these from the nanofibre one. The maximum sensitivity achieved by the dense film based sensor was 42.5 mV Pa^−1^ (Supplementary Fig. 7). Slightly different to the nanofibre device, the PVDF film device had the highest voltage response to sound at 230 Hz though the response profile was similar to our nanofibre device (Supplementary Fig. 8). Apparently, the device voltage output profile was affected by device structure and material property.

### Effect of device structure

The hole in the plastic films was found to play an important part in deciding the voltage output value (Fig. 2c and Supplementary Fig. 9). Without hole, the device can generate only 20% of the voltage. When the hole was cut just on one of the plastic films, the device generated higher voltage output than the non-hole device but lower voltage output than the dual-hole one. The single-hole device with the hole faced the sound source generated higher voltage than that on the back. This can be explained by the effect of the hole on sound propagation. Direct exposure of nanofibres to sound waves facilitates to produce higher voltage output.

The hole size affected the voltage output and sensitivity. When the hole diameter was smaller than 6.4 mm, the sensitivity was lower than 110 mV Pa^−1^ (voltage output is 1.25 V). Larger hole increased the sensitivity until the diameter reached 12.8 mm. Further increasing the hole diameter resulted in decrease in the sensitivity (Fig. 2d).

Nanofibre web thickness also affected the voltage output. When the thickness increased from 20 to 40 μm, the sensitivity increased from 161 to 205 mV Pa^−1^ (output voltage from 1.81 to 2.29 V). Further increasing the fibre web thickness led to decrease in both sensitivity and voltage output (Fig. 2e).

The nanofibre web size showed an effect on the output voltage and sensitivity. When the hole size was maintained unchanged, the output voltage and sensitivity showed a linear increase with increasing the nanofibre web size (Fig. 2f), indicating that the nanofibres covered with the plastic films also contribute to converting sound waves into electricity. Therefore the plastic films should have an effect on sensor performance. A systematic understanding on the effect of plastic films on the acoustic-to-electric conversion of PVDF nanofibre web will be reported in our future publication.

### The effect of PVDF nanofibres

The above results were based on randomly orientated PVDF nanofibre web with fibre diameter of 310±60 nm. It was expected that the fibre diameter and orientation affected the acoustic-to-electric conversion. To examine the effect from fibre diameter, we prepared randomly orientated PVDF fibre webs with different average fibre diameters (from 220 to 770 nm, see fibre morphology and diameter distribution in Supplementary Fig. 10). The fibres with average diameter of 220 and 270 nm contain beads, while all the others are bead-free fibres. Indeed, fibre diameter influenced sensor behaviour. Although all the fibre webs showed acoustic-to-electric conversion ability, their piezo-potential varied. Under the sound of frequency below 500 Hz, the bead-free fibres with the smallest diameter (310 nm) had higher piezo-potential than the others (Supplementary Fig. 11). With the same fibre web thickness and device structure, the device made of the finest non-bead nanofibres showed the highest sensitivity. The sensitivity decreased with increasing the average fibre diameter. However, the devices made of the beaded fibres had lower sensitivity, though their average fibre diameter was smaller than that of the bead-free fibres.

We also measured the β-crystal phase content of the fibres, and noted that the β-phase content changed with fibre average diameter. The change of β-phase content with fibre average diameter followed a similar trend to the change of device sensitivity with average fibre diameter. This suggests that the piezo-potential is mainly controlled by the β-crystal phase of nanofibres. This result is in accordance with our previous report on the effect of PVDF fibre diameter on mechanical-energy-to-electricity conversion^25^.

Apart from randomly orientated fibre web, aligned PVDF nanofibre mat was also prepared. With similar fibre diameter, the aligned nanofibre web showed even higher electric outputs than the randomly oriented ones (see fibre morphology and output result in Supplementary Fig. 12). It was also noted that the aligned PVDF nanofibres had higher β-phase content (90%) than the randomly orientated nanofibres (86%).

### Detection of multiple sound waves

**Figure 3:**
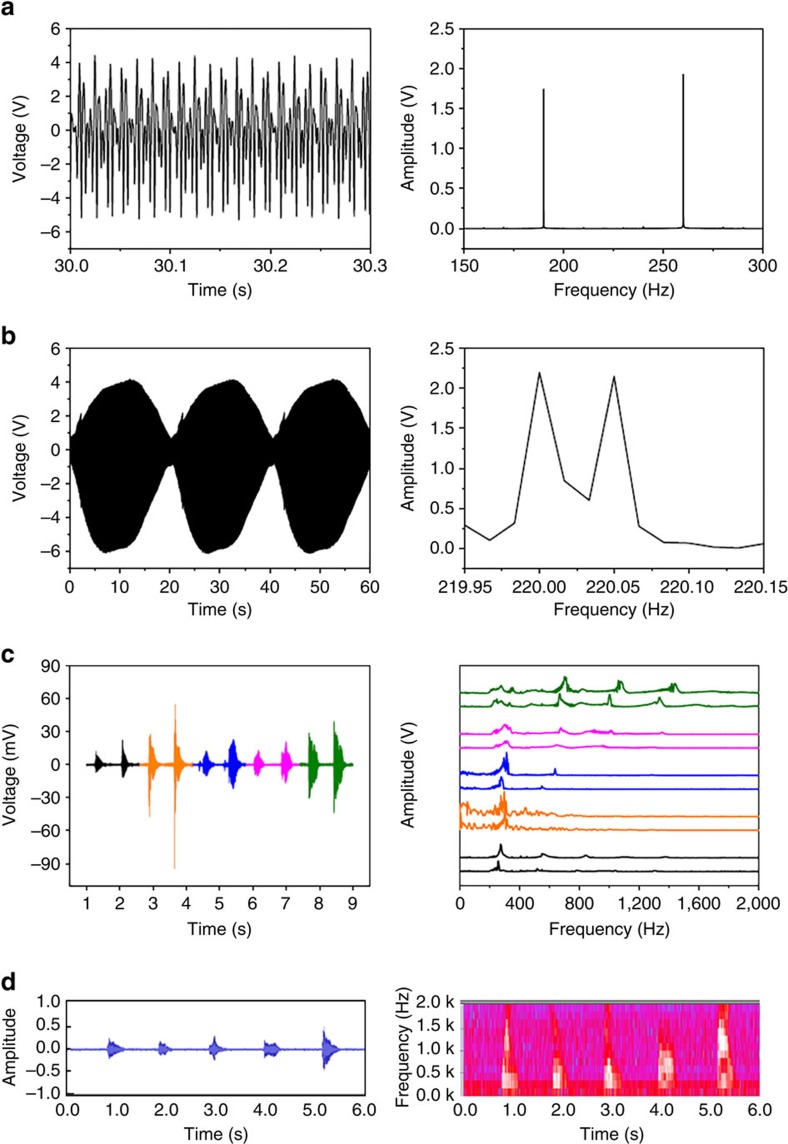
Sensing resolution and sound detection. Voltage outputs of the nanofibre sensor device under bi-frequency sound waves and the FFT-processed frequency spectrum (**a**) 190 and 260 Hz, (**b**) 220.00 and 220.05 Hz (SPL 115 dB for both loudspeakers); (**c**) voltage outputs of people's voice ‘one, two, three, four, five' and the FFT-processed frequency spectrum (SPL, 70–80 dB). The black, orange, blue, pink and green lines represent voltage outputs (left) and FFT profiles (right) of words ‘one, two, three, four, five', respectively. (**d**) Sound waveform of the same voice recorded by a commercial microphone and the FFT-processed spectrogram (the colour from blue, pink to red and white in the spectrogram indicates sound intensity increases).

The ability to differentiate multiple frequencies of sound is critical for acoustic sensors. To prove this ability, two sound acoustic sources were used to generate bi-frequency sound waves (see the setup in Supplementary Fig. 13). Under two sound waves (190 and 260 Hz), the sensor device showed a voltage output with two amplitude peaks at 190 and 260 Hz after the FFT treatment (Fig. 3a). Similarly, the nanofibre device was also able to distinguish two sound waves with very closer frequencies (220.00 and 220.05 Hz), revealing the excellent detection resolution (Fig. 3b and Supplementary Fig. 14).

To prove the usability of the nanofibre sensors, we used a nanofibre device to record people's voice. During testing, the words ‘one, two, three, four, five' were pronounced twice (louder in the second). Figure 3c shows the raw and the FFT-processed output data (also see the Supplementary Movie 1). Different words resulted in different output profiles. For comparison, the voice was also recorded by a laptop microphone and analysed by software, which showed the similar output profile (Fig. 3d). The nanofibre device has potential for sound recording.

### Comparison with other nanofibre materials

Apart from PVDF, we also tested nanofibres made of other piezoelectric polymers such as polyacrylonitrile, PVDF-HFP and P(VDF-TrFE) and non-piezoelectric polymer such as poly(vinyl alcohol). With the same device structure, these piezoelectric nanofibre devices had even higher sensitivity than the PVDF nanofibre device (Supplementary Fig. 15). The highest sensitivity was 750 mV Pa^−1^, for P(VDF-TrFE) at 100 dB. This suggests that the high acoustic-to-electric conversion property of electrospun piezoelectric nanofibres is general, suitable for various piezoelectric polymers. However, the device made of poly(vinyl alcohol) nanofibre web produced only negligible signals (Supplementary Fig. 16).

In addition, dense PET film, polyester film and cellulose paper were used separately to replace the PVDF nanofibre web in the sensor device. As expected, the replacement of piezoelectric nanofibres with those non-piezoelectric materials resulted in disappearance of the piezo-potential, and only negligible signal presumably from the vibration of the testing system was recorded (Supplementary Fig. 16).

## Discussion

It is known that polymer nanofibres are flexible and they are easy to vibrate under acoustic waves^31^. However, fibre vibration caused by sound absorption is often localized, and fibre deformations in this case differ to those caused by compression or bending of a fibrous web because the mechanical deformation in the latter case spans the whole fibrous structure. Under sound waves, the plastic films in our sensor device also vibrate and this enhances the nanofibre vibration in the plastic film covered areas.

**Figure 4:**
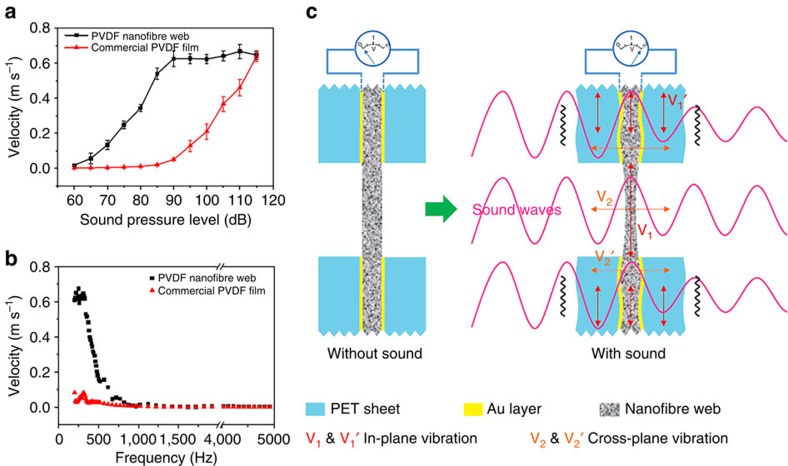
Vibration velocity and proposed piezoelectric conversion mechanism. Vibration velocity of PVDF nanofibre web and commercial PVDF piezoelectric film in the central part at (**a**) different SPLs and (**b**) different sound frequencies (SPL, 85 dB), (**c**) proposed sound vibration modes and piezoelectric conversion mechanism for the nanofibre device (the error bars represent the s.d. obtained from the test results of at least three replicates).

The vibration velocity of our PVDF nanofibre web and a commercial piezoelectric PVDF film (both 40 μm in thickness) in the central part of the hole was measured at different SPLs. As shown in Fig. 4a, when the SPL increased from 60 to 90 dB, the nanofibre web vibration velocity increased. However, the vibration velocity was stabilized at a constant level when the SPL increased to the range of 90–115 dB, suggesting the saturated vibration state under sound. In comparison, the commercial PVDF film showed a completely different vibration feature under sound. The film showed almost no vibration when the SPL was below 85 dB. However, the vibration increased gradually to the same level of the nanofibre web, when the SPL increased to 115 dB. Figure 4b shows the effect of acoustic wave frequency on the vibration of the nanofibre web and the commercial PVDF film. At 85 dB, the nanofibre web had stronger vibration than the PVDF film when the sound wave frequency was below 800 Hz, and the vibration at higher sound wave frequencies was equally weak.

A finite element method (COMSOL Multiphysics 3.5a) was also used to analyse the vibration of nanofibre web and commercial film in the central part (Supplementary Fig. 17a,b). The modelling results indicated that the vibration velocity of the PVDF nanofibre web in the central part was much higher than that of the commercial PVDF film at the SPLs and sound frequencies studied. The calculated vibration velocity for the commercial PVDF film showed a similar trend to the experiment result (Supplementary Fig. 17c,d). For the PVDF nanofibre web, the modelling result on vibration matched well with the experiment measurement only when SPL was lower than 90 dB. At the SPL above 90 dB, the calculation value of the vibration velocity showed a continuous increase with increasing SPL, which differed from the experiment value. This discrepancy between the modelling result and the experimental data can be attributed to the simplified modelling conditions and the complexity of nanofibre vibration.

Therefore, the acoustic-to-electric energy conversion of our nanofibre devices should come from the co-action of the piezoelectric electrospun nanofibres and the plastic electrodes. Figure 4c schematically illustrates the proposed sound sensing mechanism. When acoustic waves hit the piezoelectric nanofibres, vibration takes place on the fibres and plastic films because of the sound absorption. The through hole in the electrodes only allows a part of nanofibres to directly expose to sound waves. Under sound, the nanofibre web could vibrate in the in-plane (V_1_) and cross-plane (V_2_) directions. In the meanwhile, the nanofibres that are covered by the gold-coated plastic sheet also vibrate with the plastic electrodes both in-plane (V_1_′) and cross-plane (V_2_′) as well. The directly exposed nanofibres vibrate more intensively than those covered with the electrodes. Such asymmetric vibrations on the fibres and vibration propagation along the fibres enhance nanofibre deformation.

The piezoelectricity of a nanofibre web that is prepared by electrospinning of a piezoelectric polymer has been reported by our group and other researchers^16^,^17^,^21^. The high β crystal phase content and oriented macromolecular chain dipoles, in the direction across the web thickness as proposed^25^, facilitate piezoelectric conversion under compressive impact. The cross-plane vibrations (V_2_ and V_2_′) could lead to generation of piezoelectric power under acoustic waves. The in-plane vibrations assist vibration propagation throughout the entire nanofibre web.

In conclusion, we have proved the acoustic-to-electric energy conversion property of piezoelectric nanofibres and their interesting application as a transducing medium for making acoustic sensors. The sensor devices show much higher sensitivity than commercial piezoelectric PVDF film. They also have an incredible ability to distinguish sound waves in the low to middle frequency region. Since the nanofibre device shows higher sensitivity to the SPL above 100 dB, it is especially useful for detection of noise. Electrospun piezoelectric nanofibres may be useful for developing novel acoustic sensors for various applications.

## Methods

### Electrospinning

PVDF solution (20%) was prepared by dissolving 2 g PVDF pellets (*M*_W_ 275,000 g mol^−1^, Sigma-Aldrich) in 10 ml solvent mixture of dimethylformamide (Sigma-Aldrich) and acetone (Sigma-Aldrich) (volume ratio of dimethylformamide and acetone, 4/6) and heated at 70 °C for 2 h. To examine the effect of fibre diameter on energy conversion, PVDF nanofibres with different diameters were prepared from PVDF solutions with the concentration ranging from 16 to 28%. The homogeneous PVDF solution was placed to a plastic syringe with a metal needle (21 gauge). A syringe pump (KD Scientific) was used to control the flow rate at 1 ml h^−1^ and 15 kV high voltage was applied to the syringe needle using a high-voltage power supply (Gamma High Voltage). A grounded rotating drum collector (100 r.p.m.) placed 15 cm away from the needle tip was used for collecting the PVDF nanofibres.

### Sensor fabrication

A piece of electrospun PVDF nanofibre web was clamped by two gold-coated plastic films. A hole (diameter=12.8 mm) was cut in the middle of each plastic film.

### Characterizations

The morphology of nanofibres was observed under a scanning electron microscope (Zeiss Supra 55VP), and fibre diameter was measured based on the scanning electron microscope images using image processing software (ImageJ 1.45s). XRD was recorded on a diffractometer (Panalytical XRD) with Cu-Ka radiation operated at 40 kV and 30 mA. The sample was scanned in the *2θ* range of 10° to 60°. FTIR spectra were measured on a FTIR spectrophotometer (Bruker Optics) in ATR mode. Vibration velocity was measured using a CLV-2534 compact laser vibrometer.

### Test of sensor behaviour

The acoustic sensors were tested using a commercial PC speaker (Logitech, LS21) as sound source. The sound frequency was controlled by a computer and an Audio Tester software. A sound level metre (DIGITECH, QM-1589) was used to record the acoustic wave pressure. An electrochemistry working station (e-Corder 401) was used to record electric signals with a sampling frequency of 10,000 per second. To avoid false signals generated from the vibration of the testing equipment and mechanical components other than the nanofibre web, the testing equipment and the sensor device were mounted on a heavy slabstone base.

### Finite element method (FEM) modelling

The vibration mode was calculated by a finite element method, using the Pressure acoustic and Solid mechanics interaction modulus in COMSOL Multiphysics 3.5. The FEM analysis used an integrated device structure model (Supplementary Fig. 17a) consisting of two PET films and a layer of PVDF as active layer. The geometries were set the same as the actual values. Due to the complexity of nanofibrous structure, the PVDF nanofibre web was simplified into a low-density film with the density of 450 kg m^−3^. The commercial PVDF film and PET film have a density of 1,780 kg m^−3^ and 1,380 kg m^−3^, respectively. The Young's modulus of the simplified nanofibre web, dense PVDF film and PET films were set at 1,450 and 500 MPa, and Poisson's ratio 0.42, 0.35 and 0.35, respectively.

## Additional information

**How to cite this article:** Lang, C. *et al*. High-sensitivity acoustic sensors from nanofibre webs. *Nat. Commun.* 7:11108 doi: 10.1038/ncomms11108 (2016).

## Supplementary Material

Supplementary Information Supplementary Figures 1-17

Supplementary Movie 1 A video to illustrate the generation of electrical signals when different words are spoken to our sensor device.
