# Supplementary material for: High-sensitivity acoustic sensors from nanofibre webs
Source: Nat Commun. 2016 Mar 23;7:11108. doi: 10.1038/ncomms11108 (PMC4814578; doi:10.1038/ncomms11108)
Supplement: Supplementary Information — Supplementary Figures 1-17 [file ncomms11108-s1.pdf]

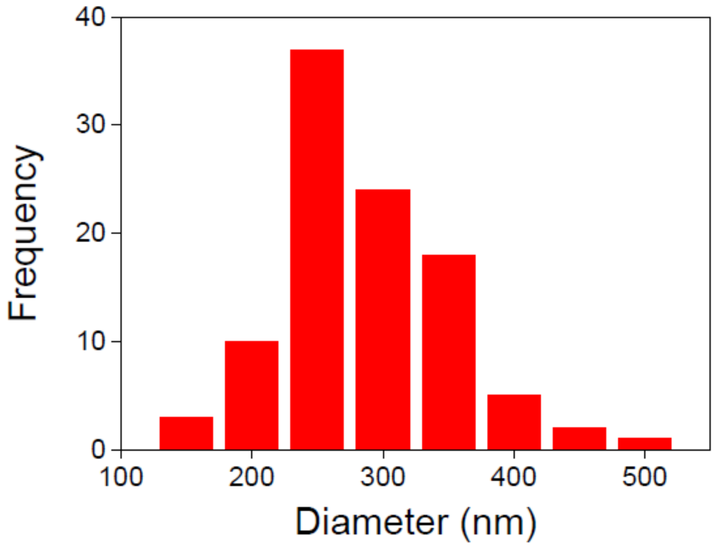

Supplementary Figure 1 | Histogram of PVDF fiber diameter distribution (nanofibers electrospun from 20% PVDF solution).

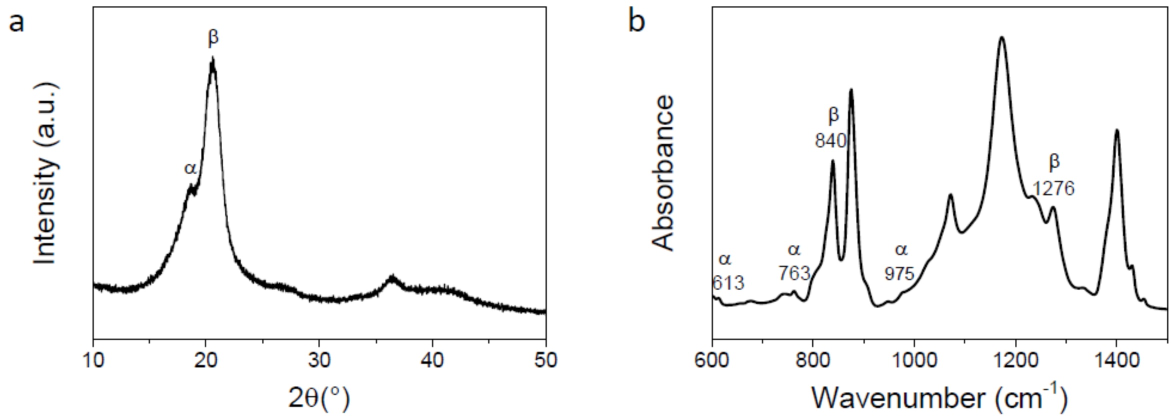

Supplementary Figure 2 | (a) XRD pattern and (b) FTIR spectrum of the PVDF nanofiber web. The X-ray diffraction pattern showed two typical peaks at 2θ = 18.4° and 20.2°, corresponding to α and β crystal phases of PVDF, respectively. The peak at 20.2° had obviously higher intensity than that at 18.4°, indicating higher β crystal phase content in the PVDF nanofibers. The FTIR spectrum showed characteristic vibration bands designated for the PVDF β phase at 840 cm^{-1} (CH_2 rocking and CF_2 asymmetrical stretching) and 1276 cm^{-1} (C-F stretching vibration). In contrast, the characteristic bands associated with the α crystal phase were found at 613 cm^{-1} (CF_2 bending and CCC skeletal vibration), 763 cm^{-1} (CH_2 in-plane or rocking) and 975 cm^{-1} (CH_2 twisting). Based on the FTIR result, the β phase content ($F(\beta)$) can be calculated using the equation, $F(\beta) = A_\beta/(1.26A_\alpha + A_\beta)$. A_β and A_α are the intensity of the β phase peak at 840 cm^{-1} and the intensity of the α crystal phase peak at 763 cm^{-1}, respectively. The calculated β phase content in the PVDF nanofibers was as high as 86%.

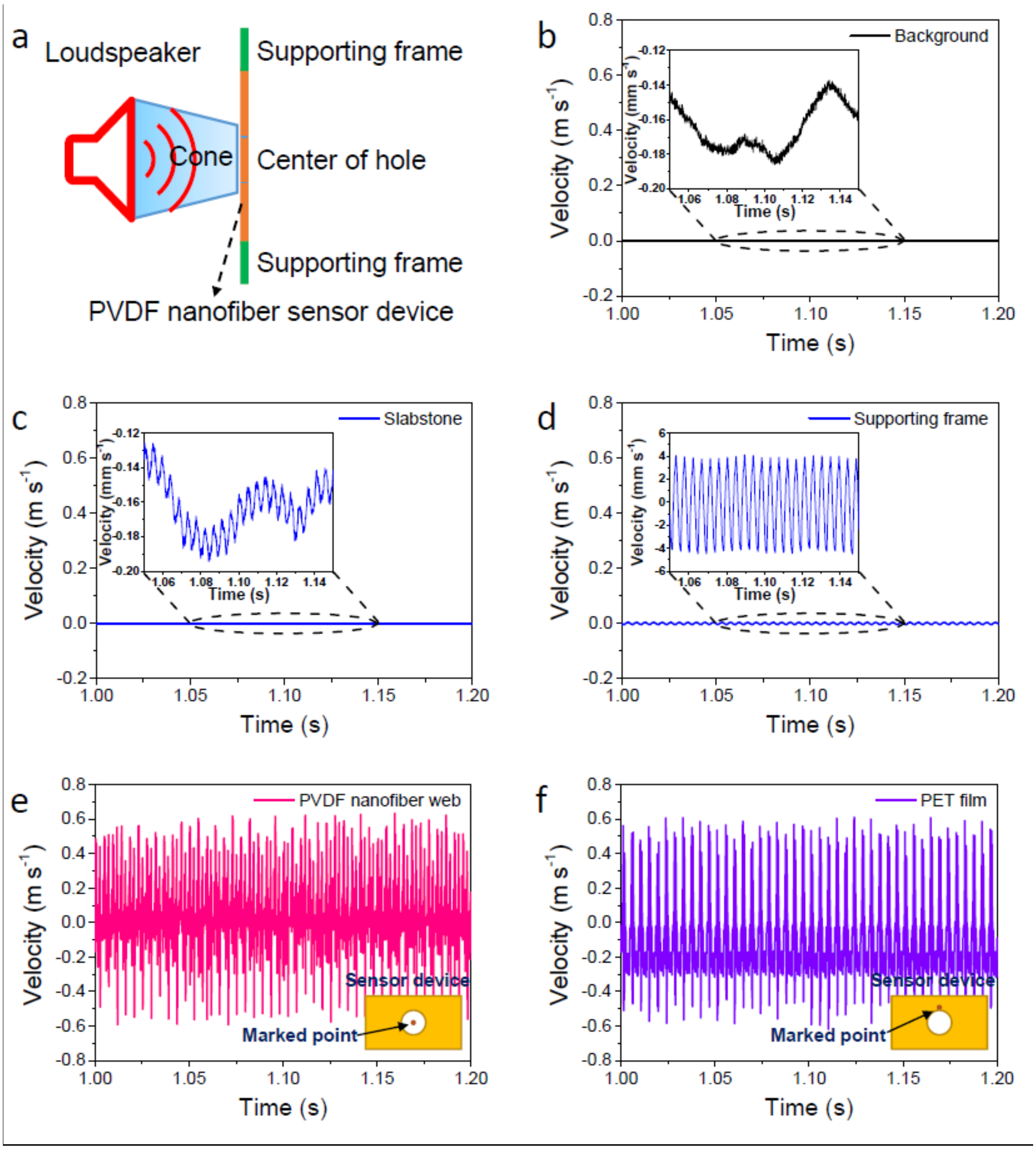

Supplementary Figure 3 | (a) Illustration of the setup to make the sound-collecting cone face the nanofiber web, (b) background vibration velocity of the sensor device (i.e. under no sound); (c) - (f) vibration velocity of (c) the slabstone, (d) the supporting frame, (e) the nanofiber web at marked point and (f) the PET film at marked point. (Sound wave frequency, 220 Hz; SPL, 115 dB) The vibration intensity of the slabstone base was almost ignorable. Its vibration during sound testing (SPL 115 dB) was equivalent to that caused by the background environment. The vibration velocity of clamps was measured as about 4 mm s^{-1}, which was much lower than that of the nanofiber web (about 500 mm s^{-1}, Supplementary Figure 3e) and the plastic film electrode (about 500 mm s^{-1}, Supplementary Figure 3f). These results indicate that the mechanical vibrations from background, supporting base and frame had negligible contribution to the voltage output of the sensor device.

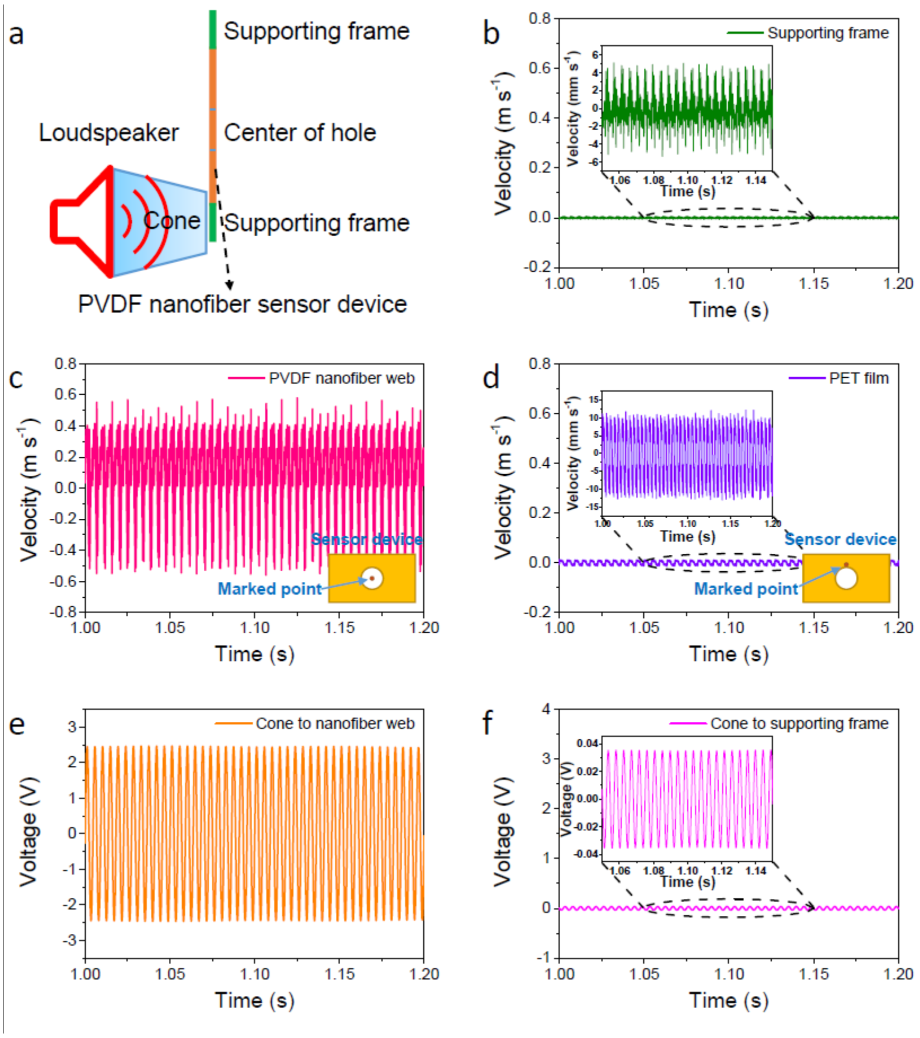

Supplementary Figure 4 | (a) Illustration of the setup for making the sound-collecting cone face the supporting frame; (b) - (d) vibration velocity on (b) the supporting frame, (c) the nanofiber web at marked point and (d) the PET film at marked point. Output voltage of the sensor device by facing the sound-collecting cone to (e) the nanofiber web and (f) the supporting frame. To rule out the electric signal caused by the vibration of the supporting frame, we adjusted the position of the sound-collecting cone to allow the sound to point to the supporting frame (Supplementary Figure 4a). Under the sound pressure level of 115 dB (Frequency, 220 Hz), the vibration velocity on the clamp was still 4 mm s^{-1} (Supplementary Figure 4b). In this case, the nanofiber web and the plastic film electrode vibrated at a velocity of 500 mm s^{-1} and 10 mm s^{-1}, respectively (Supplementary Figure 4c and Supplementary Figure 4d). The piezo-potential was below 0.04 V (Supplementary Figure 4f), the output voltage was much lower than that of the device with the sound-collecting cone directly faced to the sensor device (Supplementary Figure 4e). The vibration of both nanofiber web and plastic electrode is critical in generating large piezo potential.

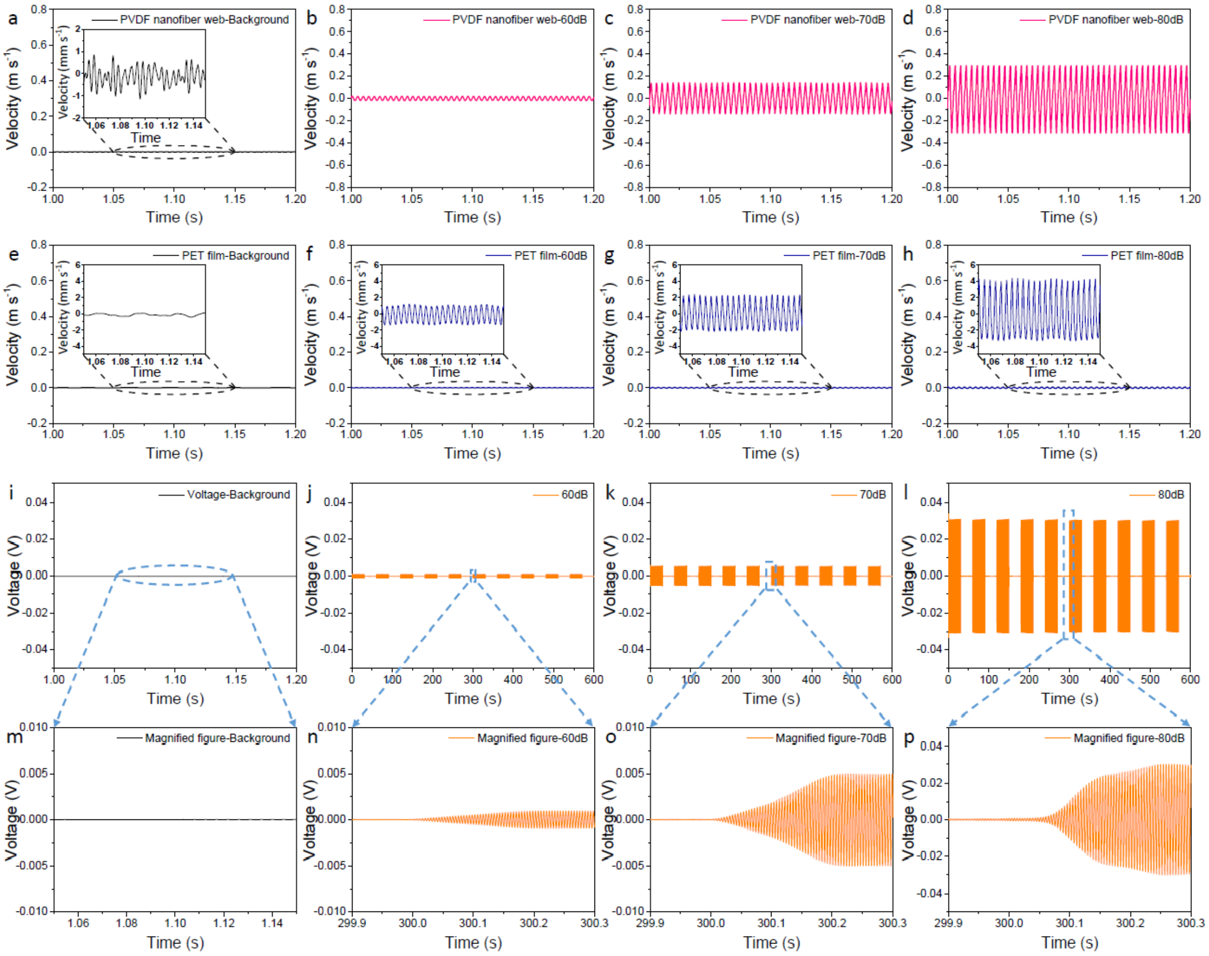

Supplementary Figure 5 | (a) - (d) Vibration velocity of the PVDF nanofiber web under background and sound of different SPLs; (e) - (h) vibration velocity of PET electrode film under background and sound of different SPLs; (i) voltage output of the nanofiber device under background, (j) - (l) voltage outputs of nanofiber device under periodic acoustic waves and background (sound on/off in every 30s), the SPL for the acoustic waves was (j) 60 dB, (k) 70 dB and (l) 80 dB.

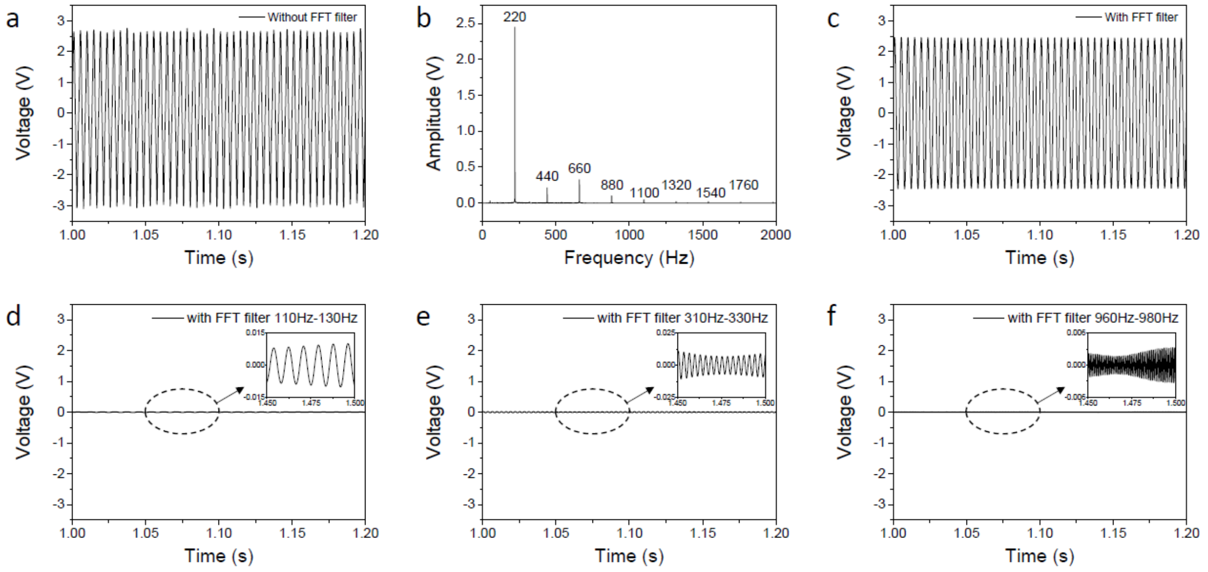

Supplementary Figure 6 | (a) Voltage output generated by the nanofiber device, (b) FFT processed frequency spectrum and (c) FFT band pass filter at 210Hz - 230Hz processed voltage output (hole diameter, 12.8 mm; thickness, 40 μm; nanofiber web size, 3×4 cm^2; sound wave frequency, 220 Hz); (d - f) processed output with FFT band pass filter at 110Hz - 130Hz, 310Hz - 330Hz and 960Hz - 980Hz, respectively. FFT is an algorithm to convert a time dependent multi-frequency signal into a frequency dependent signal. Supplementary Figure 6b shows a FFT processed frequency spectrum. The frequency of the highest voltage output matches the frequency of sound source (220 Hz). Apart from the main frequency, frequency multiplications are also observed at 440 Hz, 660 Hz, and 880 Hz. When a FFT band pass filter was processed in the range of 210 Hz - 230 Hz, the voltage signal solely occurred at 220 Hz, as shown in Supplementary Figure 6c. To verify the result, FFT filter in the range beyond the frequencies of sound source, such as 110 Hz - 130 Hz, 310 Hz - 330 Hz and 960 Hz - 980 Hz were also performed. As expected, the output was very low (Supplementary Figure 6d - 6f).

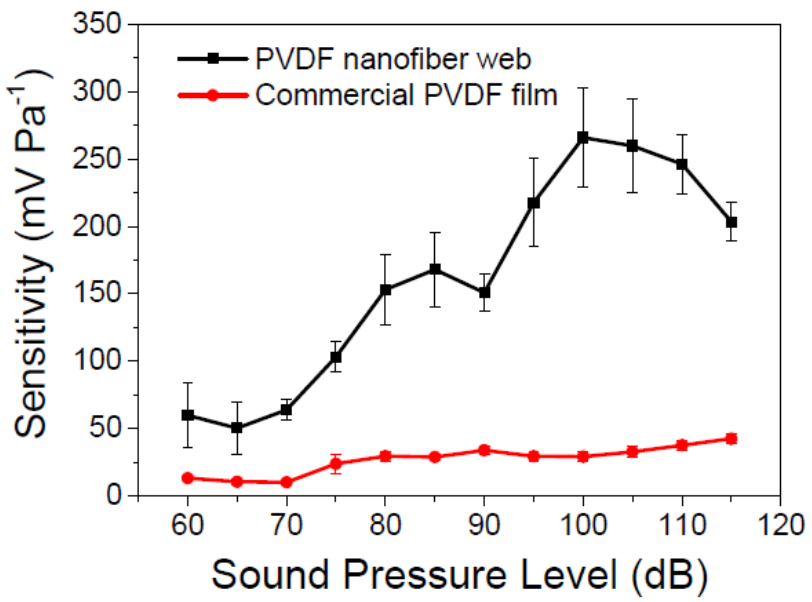

Supplementary Figure 7 | Comparison of sensitivity of nanofiber and commercial film based acoustic sensors at different sound pressure levels. The error bars represent the standard deviation obtained from the test results of at least three replicates.

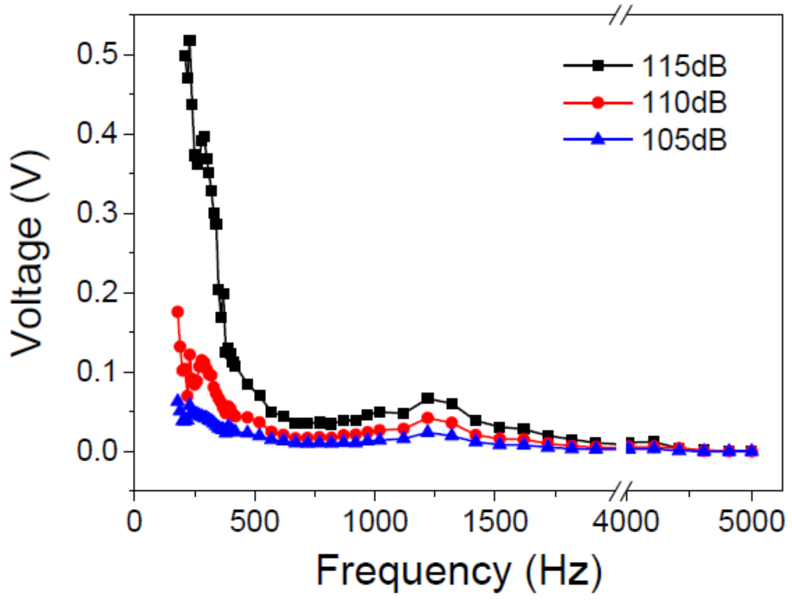

Supplementary Figure 8 | Voltage response at different sound wave frequencies for a sensor device made of a commercial piezoelectric PVDF film.

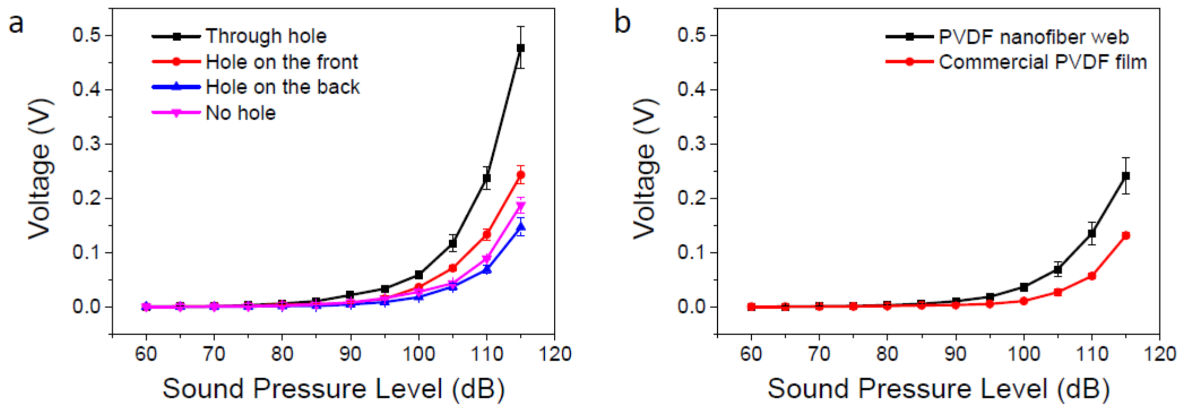

Supplementary Figure 9 | Effect of hole on voltage output, (a) sensor device made of the commercial PVDF film and (b) control sensor device with the nanofibers or film cut off in the hole area (hole diameter, 12.8 mm; nanofiber web thickness, 40 µm; nanofiber web size, 3 × 4 cm^2; sound wave frequency, 220 Hz; SPL, 115 dB). The error bars represent the standard deviation obtained from the test results of at least three replicates.

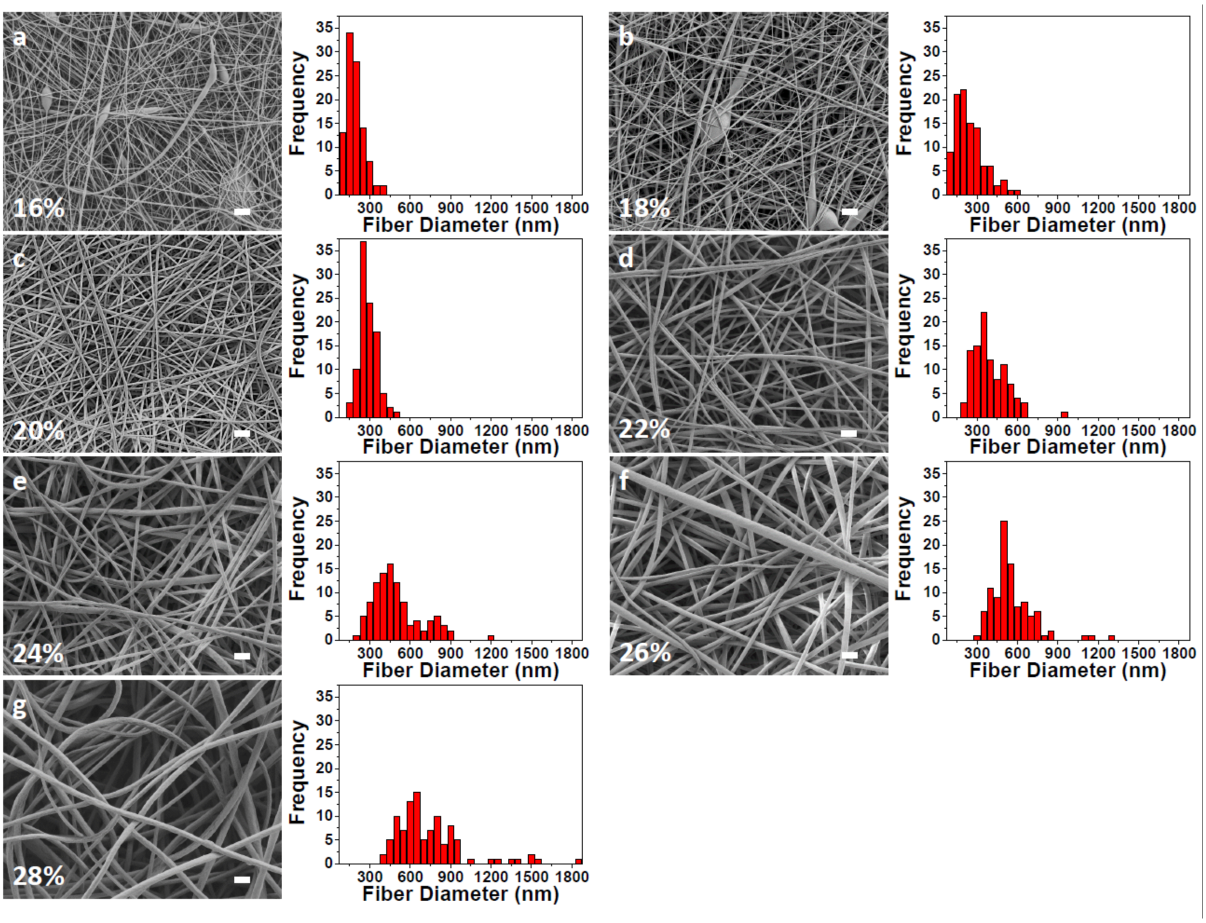

Supplementary Figure 10 | SEM image and histogram of PVDF fiber diameter distribution of the PVDF nanofibers prepared from (a) 16%, (b) 18%, (c) 20%, (d) 22%, (e) 24%, (f) 26% and (g) 28% solution. (Scale bar in SEM images, 2 μm)

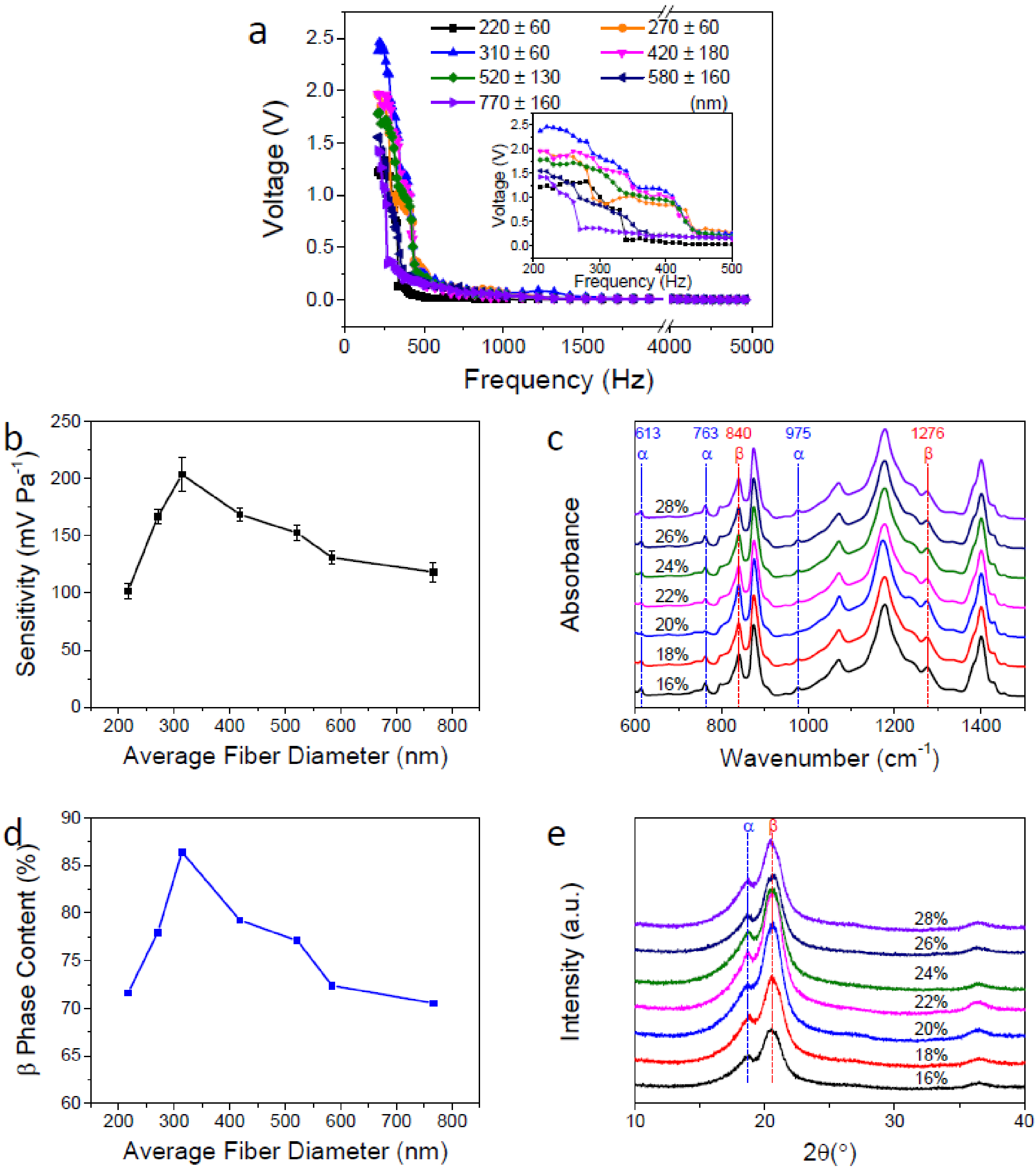

Supplementary Figure 11 | (a) The relationship between output voltage and sound wave frequency of the PVDF nanofibers with different diameters. The error bars represent the standard deviation obtained from the test results of at least three replicates. (b) Effect of fiber diameter on device sensitivity. (c) FTIR spectra, (d) *β* crystal phase content and (e) XRD patterns of the PVDF nanofibers with different diameters. (Hole diameter, 12.8 mm; nanofiber web thickness, 40 μm; nanofiber web size, 3 × 4 cm^2; sound wave frequency, 220 Hz; SPL, 115 dB)

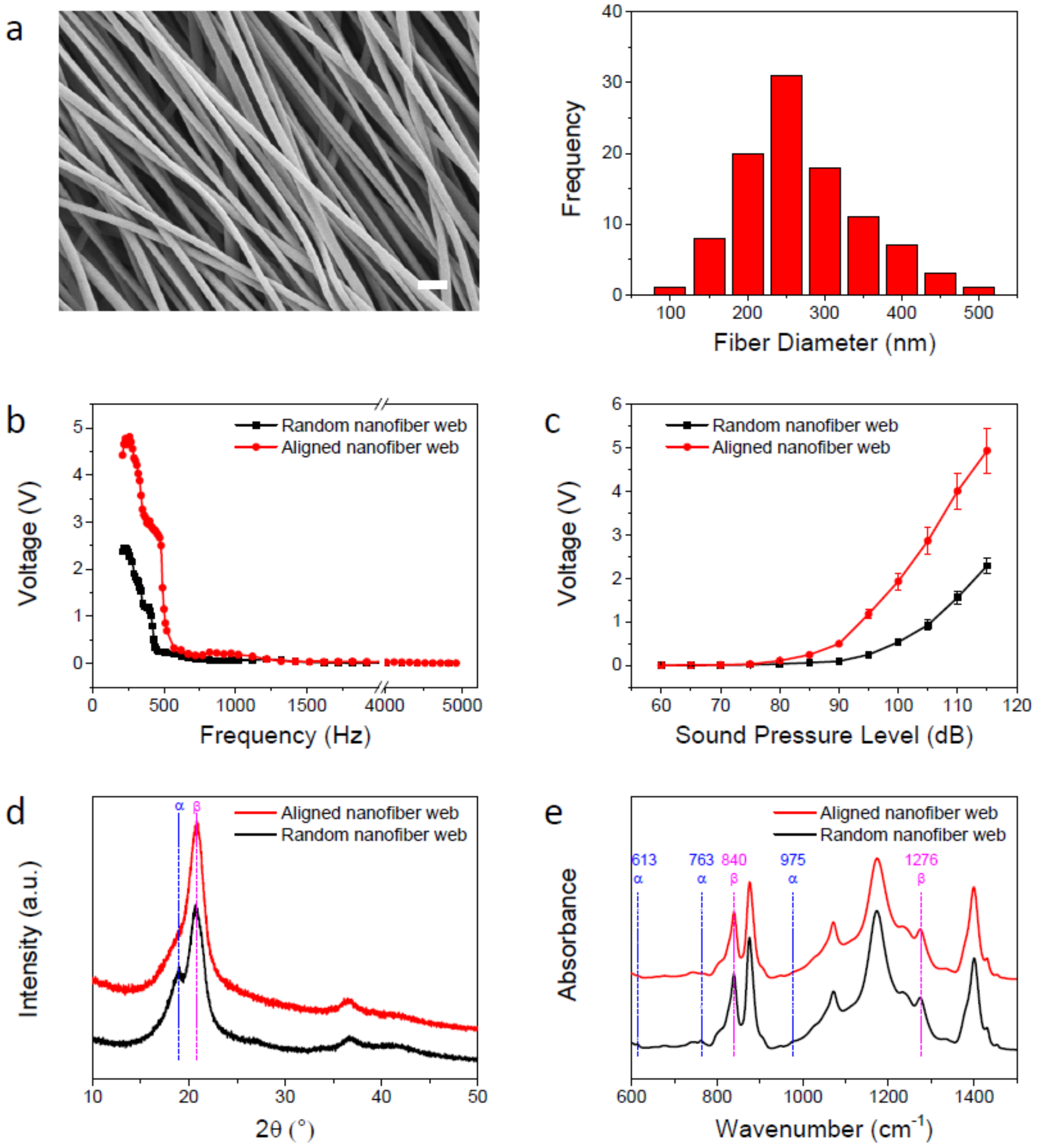

Supplementary Figure 12 | (a) SEM image and diameter distribution of aligned PVDF nanofibers (scale bar, 1 μm). Output voltage of sensor devices made from randomly oriented and aligned nanofiber web at different (b) sound wave frequencies and (c) SPLs. The error bars represent the standard deviation obtained from the test results of at least three replicates. (d) FTIR spectra and (e) XRD patterns of aligned and randomly oriented nanofiber webs. (Hole diameter, 12.8 mm; nanofiber web thickness, 40 μm; nanofiber web size, 3 × 4 cm^2; sound wave frequency, 220 Hz; SPL, 115 dB)

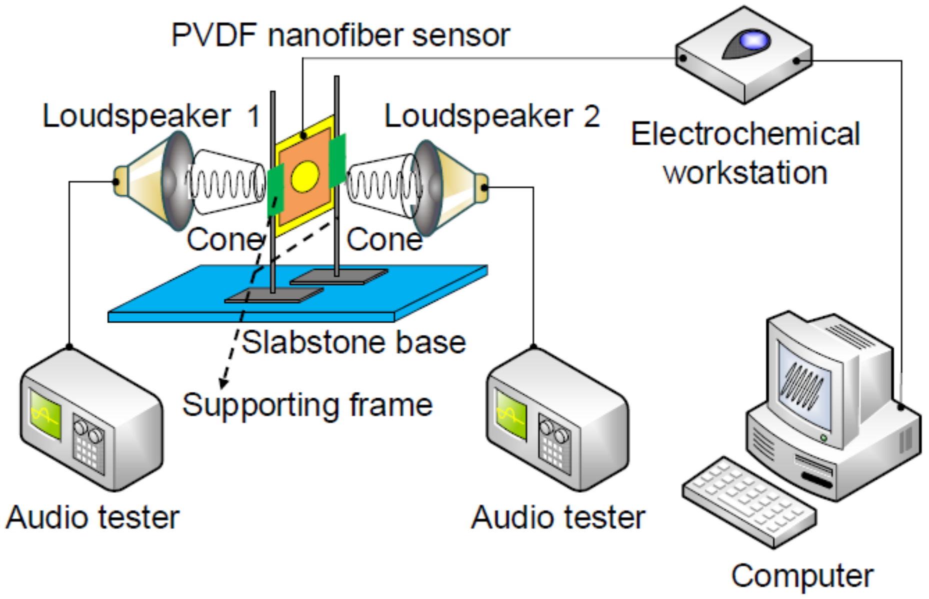

Supplementary Figure 13 | Illustration of the setup for testing bi-frequency response.

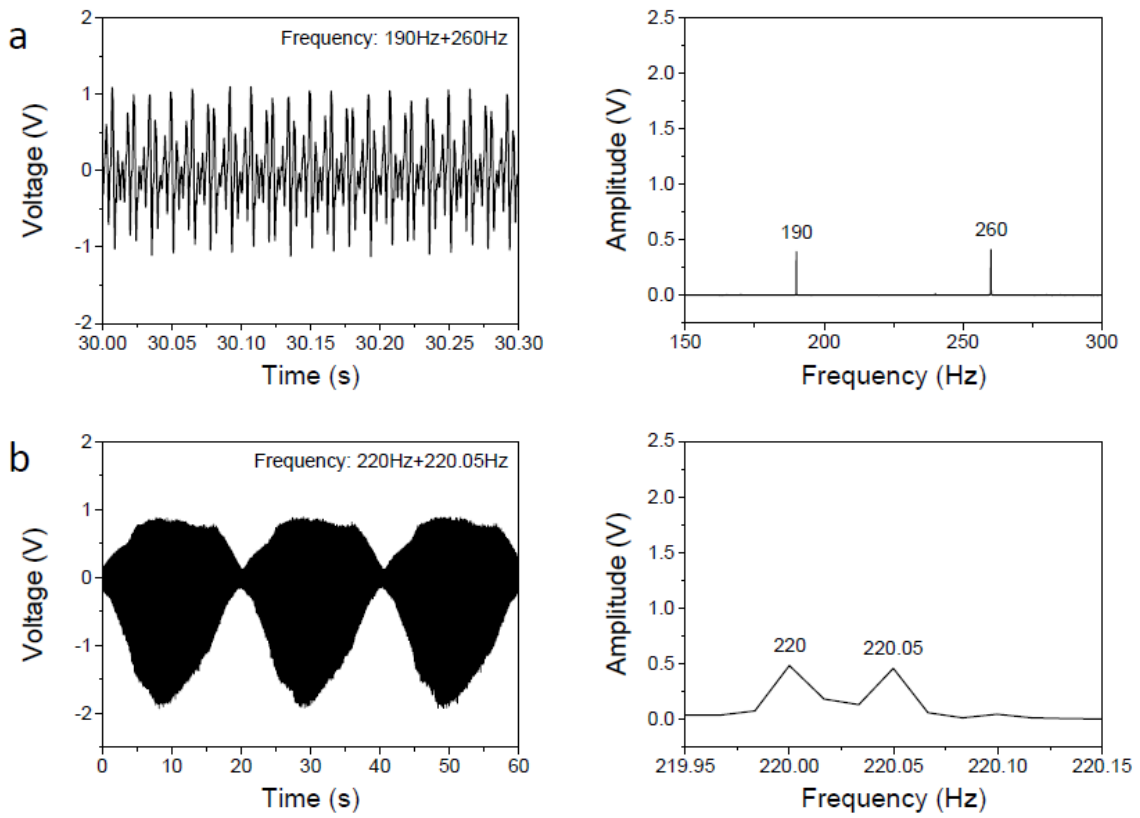

Supplementary Figure 14 | Voltage outputs of the sensor device made of the commercial piezoelectric PVDF film and the FFT processed results: (a) Sound wave frequency, 190 Hz and 260 Hz, (b) sound wave frequency, 220 Hz and 220.05 Hz (SPL 115 dB for both loudspeakers).

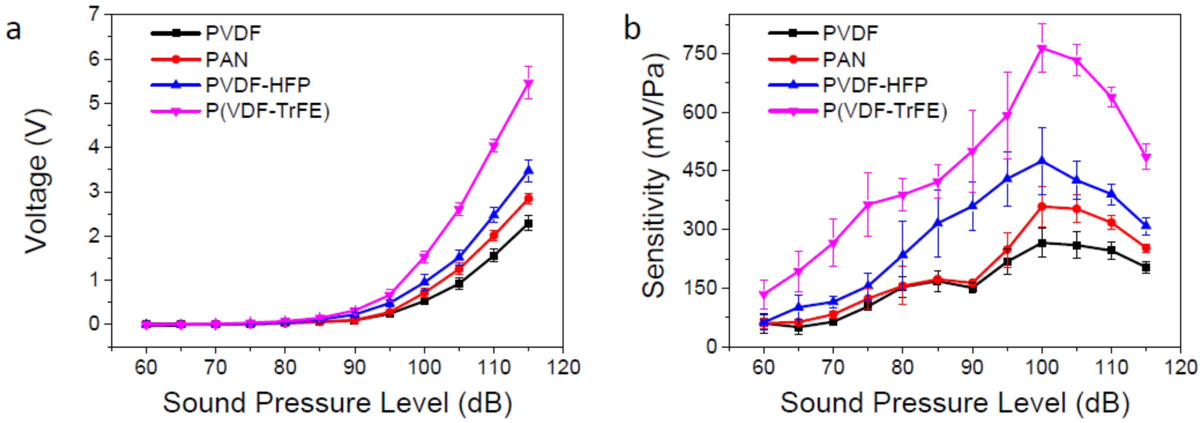

Supplementary Figure 15 | (a) Voltage outputs and (b) sensitivity of nanofiber sensors made of different piezoelectric polymers. The sensor devices from PAN, PVDF-HFP and P(VDF-TrFE) nanofiber webs have similar or even higher voltage outputs and sensitivity than the PVDF nanofiber device. The error bars represent the standard deviation obtained from the test results of at least three replicates.

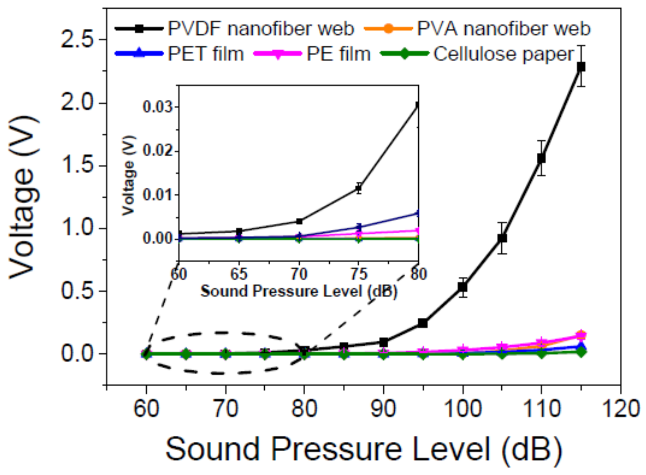

Supplementary Figure 16 | Voltage outputs of nanofiber sensors made of PVDF nanofiber web (average diameter, 310 nm; thickness, 40 μm), PVA nanofiber web (average diameter, 440 nm; thickness, 40 μm), dense PET films (thickness, 110 μm), dense PE film (thickness, 40 μm) and cellulose paper (thickness, 95 μm). The error bars represent the standard deviation obtained from the test results of at least three replicates.

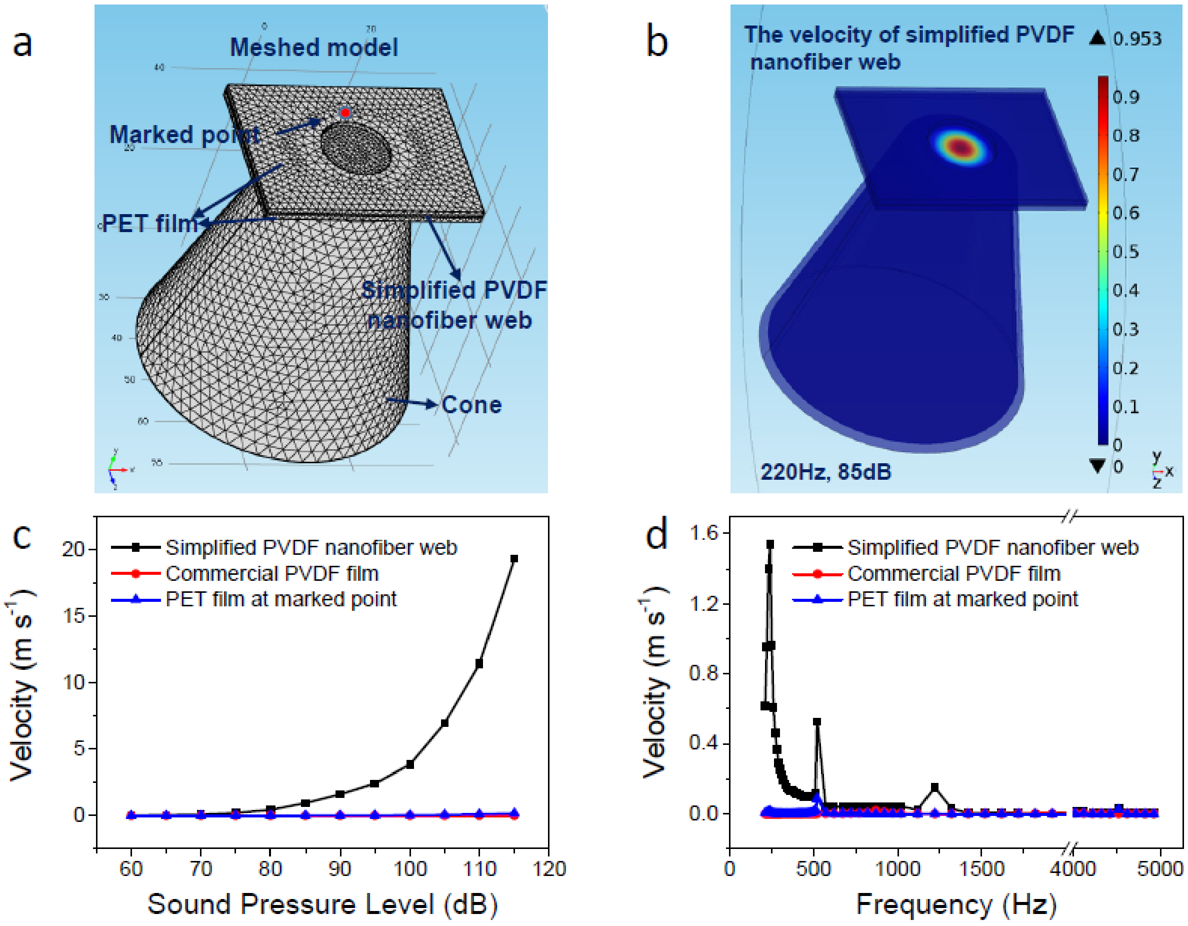

Supplementary Figure 17 | (a) Meshed model for FEM calculation, (b) - (d) FEM modeling results: (b) vibration velocity profile of the simplified PVDF nanofiber web under sound (220Hz and 85dB); Vibration velocity of simplified PVDF nanofiber web, commercial PVDF film in the central part and PET electrode film at the marked point at (c) different SPLs and (d) different frequencies. The PET films were modelled together with PVDF active layer. As expected, vibration on the PET film was much lower than that of the nanofiber web. This result is in good accordance with the experiment observation.
